# Cancer Nanomedicine

**DOI:** 10.3390/cancers12082127

**Published:** 2020-07-31

**Authors:** Clare Hoskins

**Affiliations:** School of Pure and Applied Chemistry, University of Strathclyde, Glasgow G1 1RD, UK; clare.hoskins@strath.ac.uk

**Keywords:** cancer nanomedicine, therapeutics, diagnostics, imaging, theranostics, Immunotherapy, tumour microenvironment

This Special Issue on Cancer Nanomedicine within *Cancers* brings together 46 cutting-edge papers covering research within the field along with insightful reviews and opinions reflecting our community. Cancer nanomedicine is a large umbrella under which researchers explore the physical, chemical and biological sciences. I think this is well reflected in this edition. Cancer treatments are often hindered by the lack of drug specificity, poor physicochemical properties of active pharmaceutical ingredients, poor penetration ability and drug resistance. With the discovery and characterization of an increasing number of cancer types with little improvement of the ability to diagnose, treatment options or patient prognosis, more advanced technologies are urgently required. Nanotechnology defines particulates within the 1 × 10^−9^ m range. Particulates within the nano-sized domain often exhibit unique properties compared to their larger size scale. These can be exploited in biomedicine for applications such as imaging, cell sorting, drug delivery and targeting. Cancer nanomedicine is rapidly becoming one of the leading areas of promise for cancer therapy, with first-generation treatments already available to patients.

Within this Special Issue, a diverse range of cancer nanomedicines have been discussed, including the more traditional organic-based systems, such as lipid [1,2,3,4,5,6], polymer [7,8,9,10,11] and cyclodextrin-based [12] particulates. Additionally, there are multiple studies from the growing area of inorganic systems, such as carbon nanomaterials (such as graphene oxide [13,14] and carbon nanotubes [15]) as well as other more established metallic nanomaterials, such as gold [16,17], iron oxide [18,19] and silica-based [20,21] systems. Interest into such inorganic systems has boomed over the last ten years, largely down to their multifunctional capabilities, in imaging [15], photothermal ability [22,23] or use in radiation enhancement [24]. Within this arena, a new class of nanoplatform has also developed, which is gaining traction. These platforms can be used for combined diagnostics and therapy, known as theranostics. The theranostic community is growing rapidly and in this issue a review of theranostics under development [25] as well a scientific paper [20] have been included.

One of the major challenges in cancer nanomedicine is tumour targeting and penetration. Conjugation of surface targetingligands, peptides and other molecules are of major focus within this field [26], including the use of TAT peptides [27], vitamins such as riboflavin [28], integrins [29] and antibodies [30]. Other issues such as tumour microenvironment also contribute to such challenges, and discussion on nanomedicine uptake looking at mechanistic evaluations such as shear stress [31], a hypoxic environment [32] and in overexpressing cell lines [33] have also been included.

Rapid clearance via the immune system has been another barrier historically faced by nanotechnologies. As such, nanomedicines have been developed that are inspired by or mimetic of biological systems such as extracellular vesicles [34] and exosomes [35] that exploit the naturally occurring nano vehicles produced inside the body to extract and repurpose as drug delivery systems. Other clever systems utilise other biomolecules in order to protect their nanoparticle payload, such as cloaking with cell membranes [36]. Other systems seek to deliver biomolecules such as siRNA [37,38] or to elicit an immune response in order to combat cancer [37,38,39,40,41].

Combination therapy has shown major improvement in chemotherapy compared with monotherapy. With improved tumour retardation, reduced drug resistance and better patient prognosis. As such, nanomedicines are under development incorporating combination therapies [30,42] in the hope to further enhance the findings found in small molecule trials, with the protective capabilities of nanomedicines through targeting techniques in order to reduce the toxic side effects of the potent compounds attributed to systemic circulation.

As the benefits of nanomedicine for cancer therapy have been realised, the incorporation of such nanotechnologies has been incorporated into larger-scale macromolecular systems. One such example is in the use of microbubbles [43]. Here, the nanotechnologies are conjugated onto the microbubble surfaces and ultrasonic energy is used as a means to cavitate the tumour tissue, allowing for deeper penetration of the nanomedicines in order for them to deliver their payload at the site of need.

As many of the cancer nanomedicines under development translate further towards the clinic, investigation on reliable scale-up and manufacture is explored. One technique that is currently dominating this field, particularly in liposomal development, is microfluidics. In this issue, we highlight its use in the manufacture of folate conjugated albumin particles incorporating Cabazitaxel [44]. The highly engineered mixing techniques and continuous flow parameters make such technology ideal for the formulation of cancer nanomedicines in the large batches required for trials and beyond. Work is ongoing globally into the evaluation of whether microfluidics can be exploited for other nanomedicine development and formulation.

The exciting advances within this field have led to cancer nanomedicines already being used clinically today. Sceptics would argue that the translation of nanotechnologies into the clinic have not matched the initial hype, with opinion included on the current state of the cancer nanomedicine field [45]. I believe, moving forward, more and more commercial success will be achieved. It is estimated that the global nanomedicine market will be worth USD 334 billion by 2025, with cancer nanomedicine dominating in this field. As the science develops and leads us down new avenues, the findings and their meaning are closely scrutinised and debated within the community. This issue includes 32 scientific manuscripts, 13 review articles and 1 case report reflecting the hot topics within this area [1,2,3,4,5,6,7,8,9,10,11,12,13,14,15,16,17,18,19,20,21,22,23,24,25,26,27,28,29,30,31,32,33,34,35,36,37,38,39,40,41,42,43,44,45,46].
